# Species assemblage networks identify regional connectivity pathways among hydrothermal vents in the Northwest Pacific

**DOI:** 10.1002/ece3.9612

**Published:** 2022-12-21

**Authors:** Otis Brunner, Chong Chen, Thomas Giguère, Shinsuke Kawagucci, Verena Tunnicliffe, Hiromi Kayama Watanabe, Satoshi Mitarai

**Affiliations:** ^1^ Okinawa Institute of Science and Technology Okinawa Japan; ^2^ X‐STAR, Japan Agency for Marine‐Earth Science and Technology (JAMSTEC) Yokosuka Japan; ^3^ School of Earth & Ocean Sciences University of Victoria Victoria British Columbia Canada; ^4^ Project Team for Developing Innovative Technologies for Exploration of Deep‐Sea Resources Japan Agency for Marine‐Earth Science and Technology (JAMSTEC) Yokosuka Japan; ^5^ Department of Biology University of Victoria Victoria British Columbia Canada

**Keywords:** biogeography, connectivity, hydrothermal vent, metacommunity, network

## Abstract

The distribution of species among spatially isolated habitat patches supports regional biodiversity and stability, so understanding the underlying processes and structure is a key target of conservation. Although multivariate statistics can infer the connectivity processes driving species distribution, such as dispersal and habitat suitability, they rarely explore the structure. Methods from graph theory, applied to distribution data, give insights into both connectivity pathways and processes by intuitively formatting the data as a network of habitat patches. We apply these methods to empirical data from the hydrothermal vent habitats of the Northwest Pacific. Hydrothermal vents are “oases” of biological productivity and endemicity on the seafloor that are imminently threatened by anthropogenic disturbances with unknown consequences to biodiversity. Here, we describe the structure of species assemblage networks at hydrothermal vents, how local and regional parameters affect their structure, and the implications for conservation. Two complementary networks were formed from an extensive species assemblage dataset: a similarity network of vent site nodes linked by weighted edges based on their pairwise assemblage similarity and a bipartite network of species nodes linked to vent site nodes at which they are present. Using these networks, we assessed the role of individual vent sites in maintaining network connectivity and identified biogeographic sub‐regions. The three sub‐regions and two outlying sites are separated by their spatial arrangement and local environmental filters. Both networks detected vent sites that play a disproportionately important role in regional pathways, while the bipartite network also identified key vent sites maintaining the distinct species assemblages of their sub‐regions. These regional connectivity pathways provide insights into historical colonization routes, while sub‐regional connectivity pathways are of value when selecting sites for conservation and/or estimating the multivent impacts from proposed deep‐sea mining.

## Graphical abstract

Hydrothermal vents are “oases” of biological productivity and endemicity on the seafloor that are imminently threatened by anthropogenic disturbances with unknown consequences to biodiversity. Here, we describe the structure of hydrothermal vent species assemblage networks, how local and regional parameters affect their structure, and the implications for conservation.

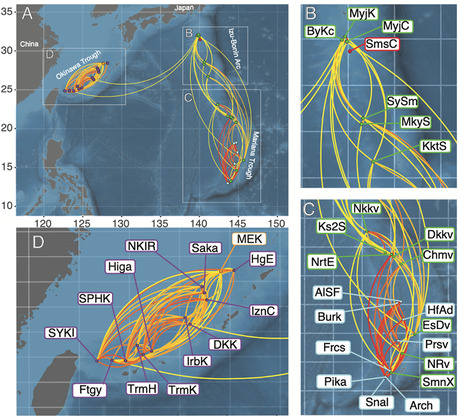

## 1 INTRODUCTION

Conservation efforts aim to slow the global degradation of biodiversity Meine et al., 2006 as well as ecosystem functions and services (Nicholson et al., 2009). These features of any ecosystem are supported by ecological connectivity, which is the flow of organisms, energy, and materials across suitable habitat patches (Crooks & Sanjayan, 2006; Correa Ayram et al., 2016). Structural or functional isolation of habitat patches by natural or anthropogenic disturbances may limit the capacity of an ecosystem to maintain processes that are valued within conservation objectives (Rudnick et al., 2012) by disrupting landscape connectivity. If the dispersal of individuals is impeded, species may become more vulnerable to global extinction by reducing their abilities to shift their ranges in response to climate change or support local recovery following disturbance events (reviewed by Jones et al., 2007). For these reasons, maintaining the structure of landscape connectivity is a global priority (IUCN, 2017). Island or “island‐like” systems (sensu (Dawson & Santos, 2016)) are particularly vulnerable to disturbances because of their characteristically isolated nature (Losos & Ricklefs, 2009; Wilson & MacArthur, 1967). Hydrothermal vents are island‐like ecosystems (Dawson & Santos, 2016; Mullineaux et al., 2018) that exhibit particularly high levels of biomass and endemicity at the seafloor (Corliss et al., 1979; Van Dover, 2014).

Hydrothermal vents are often described as “oases” of high biomass in the deep (Laubier, 1993), as local chemoautotrophy not only supports higher densities of benthic species within the vent ecosystem but also contributes to the surrounding nonvent ecosystems (reviewed in Levin et al., 2016). Vents are also home to seafloor massive sulfide deposits, a primary target for deep‐sea mining in recent years (Van Dover et al., 2018). Where these ecosystems coincide with mining interests, regional diversity may become vulnerable (Van Dover et al., 2018) and a global priority for protection (Thomas, Molloy, et al., 2021). In the Northwest Pacific, where the first test mining of hydrothermal vents occurred in the Okinawa Trough Okamoto et al., 2019, studies have assessed the impact on the local vent community and surrounding habitats at the site of the proposed mining Nakajima et al., 2015). Although over three‐fourths of vent‐endemic species in the Northwest Pacific are considered threatened by deep‐sea mining (Thomas, Böhm, et al., 2021), very few studies have attempted to assess the effect this proposed mining will have on the regional vent communities (Suzuki et al., 2018). Here we use empirical observations to describe the structure of connectivity among spatially isolated vent communities to investigate the regional impacts of deep‐sea mining.

Connectivity among vent communities is facilitated by the dispersal of planktonic larvae Adams et al., 2012. Although it is possible to quantify dispersal probabilities using oceanographic simulations (Mitarai et al., 2016), dispersal is just one of several processes required for demographic connectivity among discrete communities. Reproduction, larval dispersal, settlement, and maturation are the sequential steps necessary to maintain demographic connectivity among sites (Kritzer & Sale, 2004). Therefore, connectivity is controlled by a combination of local and regional processes such as dispersal probability, habitat suitability, and biological interactions (reviewed in Pineda et al., 2007). We investigate the drivers of diversity by formatting species' distributions of species as a network and then test how some local and regional drivers of diversity can explain the structure of this network. Such “similarity networks” have seldom been applied to empirical observations of linked but spatially distinct communities (metacommunities) (Borthagaray et al., 2015), despite a growing body of literature that has theoretically demonstrated the value of metacommunity networks to the study of biodiversity in general Keitt et al., 1997; Economo & Keitt, 2010; Suzuki & Economo, 2021).

**FIGURE 1:**
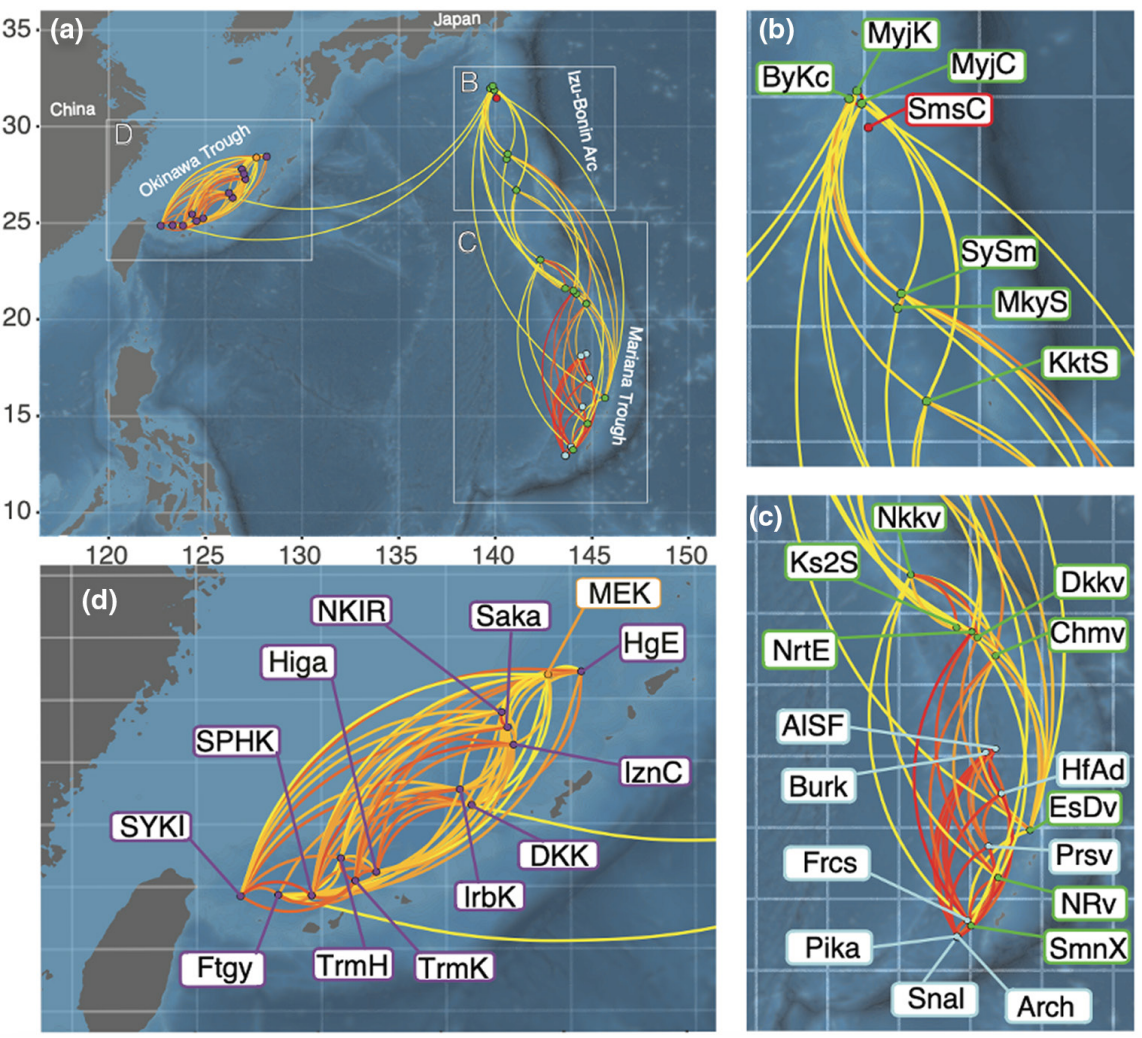
The vent sites of (a) the Northwest Pacific, (b) Izu‐Bonin, (c) Mariana, and (d) Okinawa were used in this study. The color of the vent sites represents the distinct sub‐regions as detected from the modularity analysis (purple—Okinawa Trough (OT), orange—Minami‐Ensei Knoll (MEK), red—Sumisu Caldera (SmsC), green—Izu‐Bonin‐Mariana Arc, blue—Mariana Trough (MT)). Lines connecting vent sites represent the pairwise similarity (Sorensen's coefficient) among species assemblages at vent sites. Red lines represent higher similarity while yellow lines represent lower similarity.

Previous studies have also presented hydrothermal vents as networks and applied methods from graph theory to detect biogeographic regions and infer historical connectivity pathways at the global scale (Kiel, 2016; Moalic et al., 2012). Here, we focus on similar pathways among hydrothermal vent sites of the Northwest Pacific at an intra‐regional scale more relevant to contemporary conservation. The Northwest Pacific is a distinct biogeographic region in terms of vent‐endemic fauna Bachraty et al., 2009 and dispersal through oceanographic simulations (Mitarai et al., 2016). Within this region, the interactions between the Pacific Plate, the Philippine Plate, and the Eurasian Plate create active tectonic margins containing trenches, volcanic arcs, and back‐arc spreading centres. Hydrothermal vents are present in the volcanic arcs and back arcs of the Izu‐Bonin, Mariana, and Okinawa (Figure 1). In these arc‐back‐arc basins, 78 known hydrothermal vent sites are recognized by the InterRidge database (Beaulieu & Szafrański, 2020). Although the Northwest Pacific is one of the better‐surveyed regions in terms of hydrothermal vent biodiversity, few regions are considered to have been surveyed comprehensively (Thaler & Amon, 2019). By considering the vent sites of the Northwest Pacific as an interconnected network, we can apply structural analyses from graph theory to assess the roles individual vent sites play in sharing species across the region. Maintaining connectivity through shared species within a hydrothermal vent biogeographic region is a conservation priority due to its importance to biodiversity and its vulnerability to anthropogenic disturbances (Turner et al., 2019). We use empirical observations to investigate the processes that maintain connectivity as such an approach is a recognized knowledge gap (Amon et al., 2022; Van Dover, 2014).

Hydrothermal vent species occurrence data have previously been used to detect biogeographic barriers within the Northwest Pacific (Giguère & Tunnicliffe, 2021; Kojima & Watanabe, 2015; Nakajima et al., 2014; Watanabe et al., 2019; Watanabe & Kojima, 2015). These studies have inferred connections using the number of shared species or β‐diversity between sites and/or SIMPROF analysis Clarke et al., 2008 to detect significantly similar species assemblages among vent sites and to infer the biogeographic barriers that separate others. In this study, we combined and curated the occurrence data from these previous studies along with new occurrence data to create a comprehensive view of the regional species assemblages, with representation from the three major arc‐back‐arc systems of the region. For comparability, we first replicated the same clustering methods used in the aforementioned studies. We then expanded and improved upon the previous studies by applying methods from graph theory, which offers a distinct advantage over more classical pairwise analyses of connectivity (Proulx et al., 2005) and the detection of biogeographic barriers (Bloomfield et al., 2018). As the connectivity patterns of the regional networks are inferred from shared species, they provide insights into the geological and biogeographic history of the region and the ability to identify those vent sites with a key role in maintaining the diversity structure of the region.

## 2 METHODS

### 2.1 Species occurrence data

We assembled occurrence records of vent‐associated benthic megafauna from a variety of published and new data (Table S1). The occurrence records were identified to the lowest taxonomic level with some published records being updated based on a review of recent taxonomic literature. Only species‐level records were used in this study to ensure compatibility between the different data sources and because this is the required taxonomic resolution when studying processes at the metacommunity and sub‐regional scale (Webb et al., 2003). Species names were checked against the World Register of Marine Species (WoRMS, http://www.marinespecies.org/) to ensure up‐to‐date nomenclature. All occurrences were associated with a named vent site in the InterRidge database (Beaulieu & Szafrański, 2020) based on its geographic location or associated metadata to create a site‐by‐species matrix of 36 vent fields and 117 species (Table S2). Due to the remote nature of vent ecosystems and the difficulty in carrying out comprehensive surveys, this matrix is a “presence‐only” dataset, as species absence from vent sites cannot be confirmed.

### 2.2 Similarity network

We calculated the Sørensen's coefficient (Sorensen, 1948) from the site‐by‐species matrix to give a pairwise dissimilarity between all vent sites based on their species assemblages. The Sørensen's coefficient was used as it is applicable to presence‐absence datasets, and gives extra weight to the shared presence (Legendre & Legendre, 2012). Furthermore, the Sørensen's coefficient was used to compare results with those of previous studies in this region (Kojima & Watanabe, 2015; Nakajima et al., 2014; Watanabe et al., 2019; Watanabe & Kojima, 2015), which also used this coefficient. A subsequent SIMPROF analysis (Clarke et al., 2008) used 1000 permutations at 5% significance to hierarchically group vent sites into clusters (hereafter referred to as SIMPROF clusters to avoid confusion with the defined term “network cluster” used in graph theory) that had similar species assemblages.

Using the pairwise similarity (1—Sørensen's coefficient) between vent sites, we created a network of vent sites in the Northwest Pacific. The similarity value was used as the weight of the edges that link the vent site nodes in this network, hereafter referred to as a “similarity network.” The “percolation threshold” of the similarity network was calculated following the methods of Rozenfeld et al. (2008) using the “sidier” package in R (Muñoz‐Pajares, 2013; R Core Team, 2021) and used to remove “weak” links. The relative importance of each vent site in maintaining a connected network was then evaluated based on their “betweenness centrality” (Freeman, 1977), the frequency they occur in the geodesic path between each pair of vents in the network. Betweenness centrality was calculated for every node in the similarity network after thresholding using the “igraph” package in R (Csardi & Nepusz, 2006).

We used variance partitioning to determine the contribution of environmental and spatial parameters to explain the β‐diversity (Legendre & Legendre, 2012) represented by the edge weight between nodes in the species assemblage network. The spatial parameter in question was calculated using “distance‐based Moran's Eigenvector Maps” (dbMEM) following the methods detailed in Legendre and Legendre (2012). These dbMEMs summarize the relative position of each site based on their geodesic distance from their neighboring sites. Two sets of dbMEM were created; the “fine‐scale dbMEM” uses a threshold of geodesic distance lower than that required to connect sites within the Okinawa Trough to other sites in the Northwest Pacific, while the “broad‐scale dbMEM” does consider this connectivity when calculating relative position. The environmental parameters tested were the depth and tectonic setting as recorded in the InterRidge database (Beaulieu & Szafrański, 2020) with some additional corrections (Table 1). These local environmental variables were selected because they have been recorded for every vent site in the dataset and are indicative of the many processes that directly affect local habitat suitability (Giguère & Tunnicliffe, 2021; Mullineaux et al., 2018; Tunnicliffe et al., 1998). The variance partitioning analyses and the formation of the dbMEMs were carried out using the “vegan” package in R (Oksanen et al., 2019).

**TABLE 1:** Description of vent sites used in analyses

Vent site ID	Vent site name	Lat. (°N)	Lon. (°W)	Depth (m)	Tectonic setting	Region	SIMPROF group	Module membership	Zi	Pi	BetweenessCentrality	Species richness
DKK	Daisan‐Kume Knoll	26.302	126.413	1370	arc volcano	Ryukyu Arc	4	OT	0.87	0.38	161.74	17
Ftgy	Futagoyama	24.867	123.308	1270	back‐arc spreading center	Okinawa Trough	4	OT	1.01	0.22	85.73	16
HgE	Higashi‐Ensei	28.439	128.172	1220	back‐arc spreading center	Okinawa Trough	4	OT	1.44	0.27	0.22	20
Higa	Higa	26.555	126.223	1485	back‐arc spreading center	Okinawa Trough	4	OT	0.72	0.24	1.61	14
IrbK	Irabu Knoll	25.230	124.880	1850	back‐arc spreading center	Okinawa Trough	4	OT	0.58	0.26	2.04	13
IznC	Izena Cauldron	27.267	127.083	1610	back‐arc spreading center	Okinawa Trough	4	OT	1.44	0.32	0.22	21
NKIR	North Knoll, Iheya Ridge	27.791	126.897	1100	back‐arc spreading center	Okinawa Trough	5	OT	3.60	0.37	2.04	41
Saka	Sakai	27.548	126.990	1600	back‐arc spreading center	Okinawa Trough	5	OT	3.60	0.37	2.04	41
SPHK	SPOT, Hatoma Knoll	24.855	123.841	1520	back‐arc spreading center	Okinawa Trough	4	OT	1.73	0.18	2.04	21
SYKI	SPOT, Yonaguni Knoll IV	24.849	122.700	1385	back‐arc spreading center	Okinawa Trough	4	OT	1.01	0.12	0.22	15
TrmH	Tarama Hill	25.454	124.310	1973	back‐arc spreading center	Okinawa Trough	6	OT	1.01	0.00	0.00	14
TrmK	Tarama Knoll	25.092	124.542	1990	back‐arc spreading center	Okinawa Trough	6	OT	2.02	0.16	1.61	23
MEK	Minami‐Ensei Knoll	28.392	127.642	740	back‐arc spreading center	Okinawa Trough	3	ME	3.87	0.57	0.00	27
SmsC	Sumisu Caldera	31.467	140.067	690	arc volcano	Izu‐Bonin Arc	2	Su	1.73	0.56	0.00	5
AlSF	Alice Springs Field	18.210	144.707	3640	back‐arc spreading center	Mariana Trough	1	MT	2.44	0.17	0.00	22
Arch	Archaean	12.939	143.632	3060	back‐arc spreading center	Mariana Trough	1	MT	0.31	0.18	0.00	10
Burk	Burke	18.109	144.432	3660	back‐arc spreading center	Mariana Trough	1	MT	2.05	0.10	0.00	19
Frcs	Forecast	13.400	143.917	1470	back‐arc spreading center	Mariana Trough	1	MT	1.47	0.21	189.00	17
HfAd	Hafa Adai	16.957	144.868	3294	back‐arc spreading center	Mariana Trough	1	MT	2.05	0.18	0.00	20
Pika	Pika	12.918	143.648	2990	back‐arc spreading center	Mariana Trough	1	MT	0.89	0.00	0.00	12
Prsv	Perseverance	15.480	144.508	3935	back‐arc spreading center	Mariana Trough	1	MT	0.70	0.00	0.00	11
Snal	Snail	12.953	143.620	2880	back‐arc spreading center	Mariana Trough	1	MT	1.47	0.00	0.00	15
ByKc	Bayonnaise Knoll caldera	31.967	139.733	900	back‐arc spreading center	Izu‐Bonin back‐arc	9	IBMa	0.75	0.34	9.92	10
Chmv	Chamorro volcano	20.810	144.705	896	arc volcano	Mariana Arc	11	IBMa	−0.20	0.00	0.50	5
Dkkv	Daikoku volcano	21.324	144.194	450	arc volcano	Mariana Arc	8	IBMa	1.69	0.00	0.00	11
EsDv	East Diamante volcano	15.930	145.670	457	arc volcano	Mariana Arc	8	IBMa	1.06	0.00	56.53	9
KktS	Kaikata Seamount	26.700	141.083	930	arc volcano	Izu‐Bonin Arc	7	IBMa	0.75	0.20	30.20	9
Ks2S	Kasuga 2 Seamount	21.600	143.617	500	arc volcano	Mariana Arc	8	IBMa	1.69	0.00	0.00	11
MkyS	Mokuyo Seamount	28.320	140.580	1200	arc volcano	Izu‐Bonin Arc	10	IBMa	−0.83	0.00	0.00	3
MyjC	Myojinsho Caldera	31.883	139.950	1110	arc volcano	Izu‐Bonin Arc	9	IBMa	0.75	0.40	91.26	11
MyjK	Myojin Knoll	32.103	139.868	1360	arc volcano	Izu‐Bonin Arc	9	IBMa	1.69	0.42	179.36	15
Nkkv	Nikko volcano	23.083	142.333	600	arc volcano	Mariana Arc	8	IBMa	2.01	0.00	16.08	12
NrtE	Northwest Eifuku	21.485	144.043	1604	arc volcano	Mariana Arc	11	IBMa	0.75	0.32	231.87	10
NRv	Northwest Rota‐1 volcano	14.601	144.775	599	arc volcano	Mariana Arc	11	IBMa	−0.20	0.41	0.00	7
SmnX	Seamount X	13.250	144.020	1450	arc volcano	Mariana Arc	11	IBMa	0.11	0.24	0.50	7
SySm	Suiyo Seamount	28.575	140.642	1380	arc volcano	Izu‐Bonin Arc	11	IBMa	−0.20	0.00	23.25	5

### 2.3 Bipartite network

The second network was formed directly from the site‐by‐species matrix and is referred to as a bipartite network, after the two types of nodes it contains. The first node type, a species node, is linked to the second type, a vent site node, if the species was present at said vent site. The nodes in this network are linked by unweighted edges that only occur between nodes of a different type (i.e., species—vent site). This network approach has been implemented in various biogeographic studies (Carstensen et al., 2012; Dalsgaard et al., 2014; Kougioumoutzis et al., 2014, 2017) to detect barriers to “biogeographical connectivity.” Following the methods of Carstensen et al. (2012), a simulated annealing approach was used to subdivide the regional bipartite network iteratively into groups until the grouping that maximizes the “Modularity” value of the network is identified. The “Modularity” is a measure of the extent to which nodes have more links within their group than expected if the links are random (Guimerà & Nunes Amaral, 2005a, 2005b). For this analysis, we used the “rnetcarto” package in R (Doulcier & Stouffer, 2015; R Core Team, 2021). The resultant groups of highly linked nodes in the bipartite network are hereafter referred to as “modules.” Redundancy analysis was used to detect the possible roles of known biogeographic barriers—depth, tectonic setting, and distance (broad‐scale dbMEM)—on module membership, following the recommendations of Legendre and Legendre (2012). Additionally, a MANOVA analysis was carried out with the same formula to detect any significant variation of each explanatory variable and their interacting terms between the module groups.

Each node's role in connecting the bipartite network was assessed based on its within‐module degree (z_i_) and participation coefficient (P_i_) (Guimerà & Nunes Amaral, 2005a, 2005b). As the direct links to a vent site node come from the species it contains, the position of a vent site node in z_i_ ‐ P_i_ space is indicative of its species richness, the regional distribution of those species, and the role the site itself plays in connecting spatially isolated species assemblages (Carstensen et al., 2012). Each vent node was assigned one of the universal cartographic roles defined by Guimerà and Nunes Amaral's (2005a). These roles are as follows: “Peripheral nodes” (z_i_ < 2.5 and P_i_ < 0.62), “Module hubs” (z_i_ > 2.5 and P_i_ < 0.62), “Connector nodes” (z_i_ < 2.5 and P_i_ > 0.62), and “Network Hubs” (z_i_ > 2.5 and P_i_ > 0.62). These metrics of module connectivity were calculated using the “rnetcarto” package in R (Doulcier & Stouffer, 2015).

## 3 RESULTS

### 3.1 Similarity network

**FIGURE 2:**
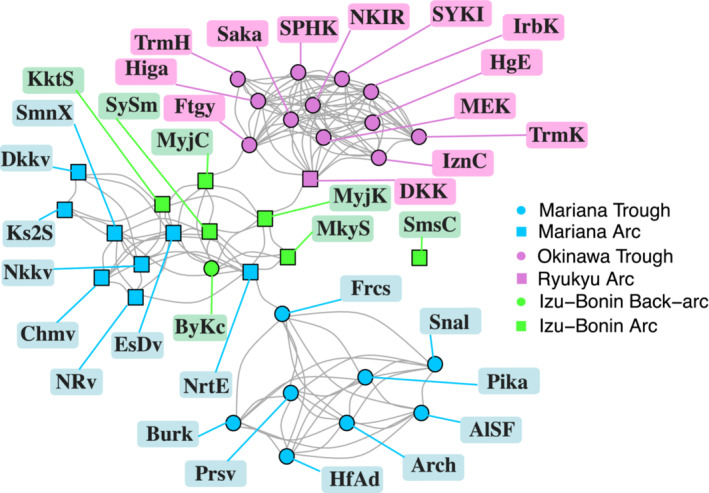
Similarity network of the Northwest Pacific vents showing qualitative clustering based on tectonic basin. The shape represents the tectonic setting of each vent site, and the color represents their basin. Edges represent pairwise similarity values among vent nodes above the percolation threshold. The relative position of the vent nodes is dictated by the shared edges.

**FIGURE 3:**
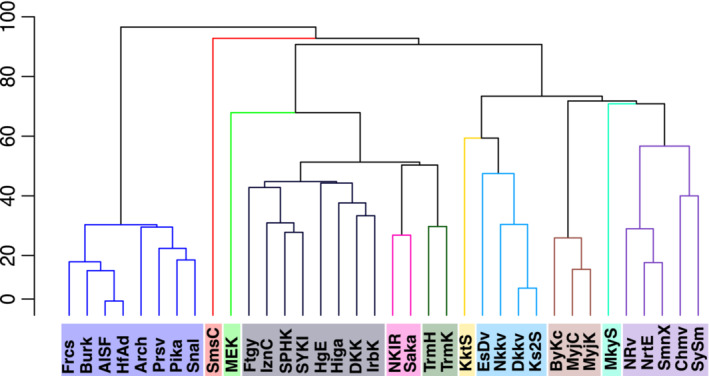
SIMPROF clustering of Northwest Pacific vent sites based on Sorensen's coefficient

The similarity network shows three distinct groups in the form of qualitative network clusters (Newman, 2010) once the percolation threshold of 0.7 was applied. The edges that remain after this threshold and how they connect vent sites can be seen in a geographic (Figure 1) or simplified layout (Figure 2). The simplified layout positions vent sites relative to others with which they are directly linked, revealing three qualitative network clusters. These three network clusters are the vent sites of the Mariana Trough, the Okinawa Trough, and the Izu‐Bonin‐Mariana Arc. The betweenness centrality of nodes (Table 1) was high for those vent sites that linked the three network clusters: Northwest Eifuku, Forecast, and Myojin Knoll. However, the SIMPROF analysis returned 11 groups (SIMPROF clusters) of vent sites each of which has no significant structural differences among their species assemblages (Figure 3).

**FIGURE 4:**
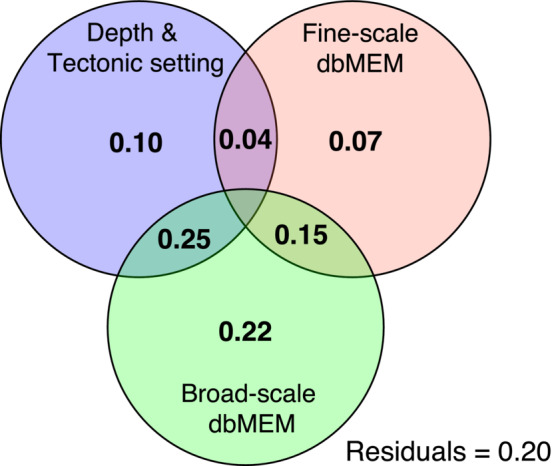
Variance partitioning of vent site dissimilarity (Sorensen's coefficient) against local environmental variables (depth and tectonic setting), fine‐scale dbMEM, and broad‐scale dbMEM. The low residuals show how well these variables predict dissimilarity, particularly the broad‐scale dbMEM.

The explanatory variables used in the variance partitioning (Figure 4) explain 80% of the variation in the Sørensen's coefficient between vent sites. Much of this variation is explained by the broad‐scale dbMEM; alone, it explains 22% while its interaction with the environmental parameters of depth and tectonic setting explains an additional 25%. Alone the environmental variation among vent sites explains 10% and a further 4% when combined with the fine‐scale dbMEM spatial parameter. The fine‐scale dbMEM only explains 7% of variation on its own and a further 15% as an interaction with the broad‐scale dbMEM. Depth was checked for spatial autocorrelation following the methods outlined in Legendre and Legendre (2012) and found to be nonsignificant.

### 3.2 Bipartite network

**FIGURE 5:**
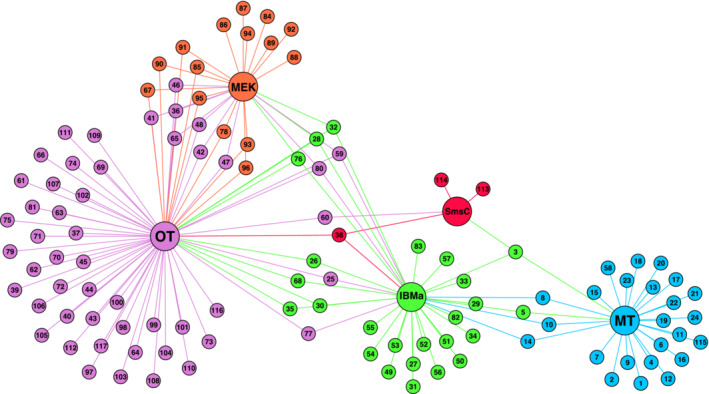
Bipartite network of Northwest Pacific with site nodes contracted by module group connected by species nodes. Node colors represent module groups; purple—Okinawa Trough (OT), orange—Minami‐Ensei Knoll (MEK), red—Sumisu Caldera (SmsC), green—Izu‐Bonin‐Mariana Arc (IBMa), blue—Mariana Trough (MT). Species nodes represent unique species, identified by number in supplemental Table S3. While no species are shared among the three sub‐regions, IBMa's role as an intermediary between OT and MT is highlighted by its shared species.

The simulated annealing method (Guimerà & Nunes Amaral, 2005a, 2005b) detected five distinct modules (Figure 5). Hereafter, we call these modules: OT (Okinawa Trough module), MEK (Minami‐Ensei Knoll site), SmsC (Sumisu Caldera site), IBMa (Izu‐Bonin‐Mariana arc module), and MT (Mariana Trough module). The OT, IBMa, and MT are hereafter collectively referred to as the sub‐regions of the Northwest Pacific while ME and SmsC are considered single‐site outliers. The species nodes within a module represent species that are more closely associated with vent sites of that module than any other; many are not found at vent sites outside of their module (module endemics). Of the sub‐region modules, the MT had the highest proportion of module endemics 80% (21/26), followed by OT with 57% (38/66), and finally IBMa with 47% (16/34) (Figure 5).

**FIGURE 6:**
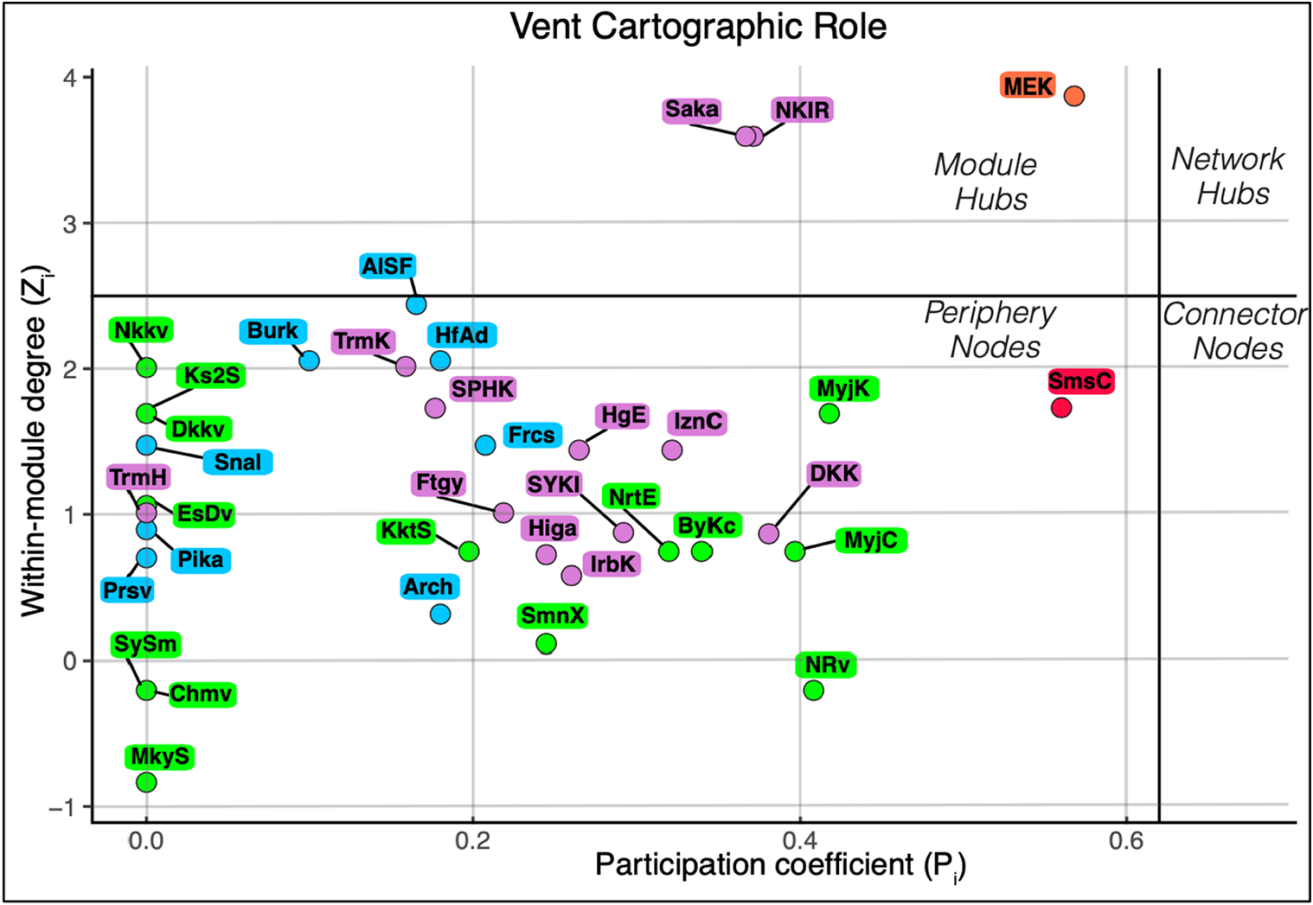
Within‐module degree and participation coefficient of each vent site in the bipartite network. Colored by module membership as in Figure 5; purple—OT, orange—ME, red—Su, green—IBMa, blue—MT.

The distribution of vent sites in terms of within‐module degree (z_i_) and participation coefficient (P_i_), as well as their category of “universal cartographic roles” (Guimerà & Nunes Amaral, 2005b), is presented in Figure 6. The universal cartographic roles of species nodes are summarized in Table S3. The vent sites with the highest within‐module degree of their respective sub‐regions are Sakai (OT), Nikko (IBMa), and Alice Springs (MT). Of the three sub‐regions, the vent site with the highest participation coefficient is Myojin Knoll of the IBMa (P_i_ = 0.42). Other than the outliers, the five vent sites with the highest participation coefficients are Myojin Knoll, Northwest Rota, and Myojinsho Caldera of the IBMa, and Daisan‐Kume Knoll and North Knoll, Iheya Ridge of the OT module. All vent sites of the MT had relatively low participation coefficients, with Forecast having the highest 0.21 The vent sites that only contain module endemics have the lowest participation coefficients of 0 and are referred to as “ultra‐periphery” nodes. There are 11 such ultra‐periphery nodes among the vent sites (Mokuyo Seamount, Chamorro volcano, Suiyo Seamount, Perseverance, Pika, Tarama Hill, East Diamante volcano, Snail, Daikoku volcano, Kasuga 2 Seamount, Nikko volcano), with representation from each of the three sub‐regions.

The explanatory variables used in the redundancy analysis (broad‐scale dbMEM, depth, and tectonic setting) were able to explain 92.8% of the module grouping, with tectonic setting and broad‐scale dbMEM varying significantly between modules (*p* < 0.01 and 0.05, respectively).

## 4 DISCUSSION

The results obtained from the two networks generated in this study generally agree on the regional diversity structure and the significance of the environmental drivers we investigated. Although both networks are formed from the same species assemblage data, they each have distinct advantages in the way they can be interpreted. The similarity network builds upon common ecological analyses but also extends traditional clustering approaches to detect sites that act as intermediaries between clusters. Furthermore, displaying β‐diversity as a network of similarity edges is arguably a much more intuitive way of visualizing species diversity and inferred connectivity. The bipartite network is less intuitive in its presentation, but it can go beyond the detection of intermediary sites between clusters and identify sites that act as hubs of shared species within their cluster (module). Previous studies have used both network methods to study other ecosystems at various scales but not in combination. The discrete nature and dispersal processes that dictate connectivity at hydrothermal vents make them particularly suitable for the application of such network methods.

### 4.1 Regional diversity structure

At the scale of the entire region, the structure of the similarity network has “small world” properties due to the presence of tight clusters of nodes with key connectivity pathways (Figure 2) between them (Watts & Strogatz, 1998). This same small world structure can be seen in the vent larvae dispersal network of Mitarai et al. (2016) and in various marine metapopulation studies that evaluated the structure of dispersal networks Kininmonth et al., 2010; Ramesh et al., 2019; Watson et al., 2011). Rozenfeld et al. (2008), who pioneered the application of network analysis to measures of pairwise site similarity, found the same small world structure using genetic similarities between populations of seagrass species.

The clustering that gives the similarity network its small world properties is evident in the spatial arrangement of nodes based on their shared edges (Figure 2) and the high betweenness centrality of the key nodes (Table 1) that connect these three network clusters (Mariana Trough, Okinawa Trough, and Izu‐Bonin‐Mariana Arc). This outcome contrasts with the 11 SIMPROF clusters (Figure 3) that represent the lowest hierarchical level at which there are no significant structural differences between the vent site assemblages (Clarke et al., 2008). At the higher hierarchical level, the SIMPROF clusters are contained within the three “super‐clusters” of Mariana Trough, Okinawa Trough, and Izu‐Bonin‐Mariana Arc as demonstrated by Kojima and Watanabe (2015). We detected the same three sub‐regions using a modularity analysis of the bipartite network with the additional presence of two outlier sites (Minami‐Ensei Knoll and Sumisu Caldera). At a glance, geographical proximity seems to be a strong driver of the module grouping (Figure 1a) with OT to the west of the region, IBMa mostly to the northeast, and MT to the southeast. It is likely that OT is spatially isolated based on the low dispersal probability connecting it to the rest of the region (Mitarai et al., 2016), but the differentiation between IBMa and MT can be better explained by the tectonic setting (Giguère & Tunnicliffe, 2021) (Figure 2).

**FIGURE 7:**
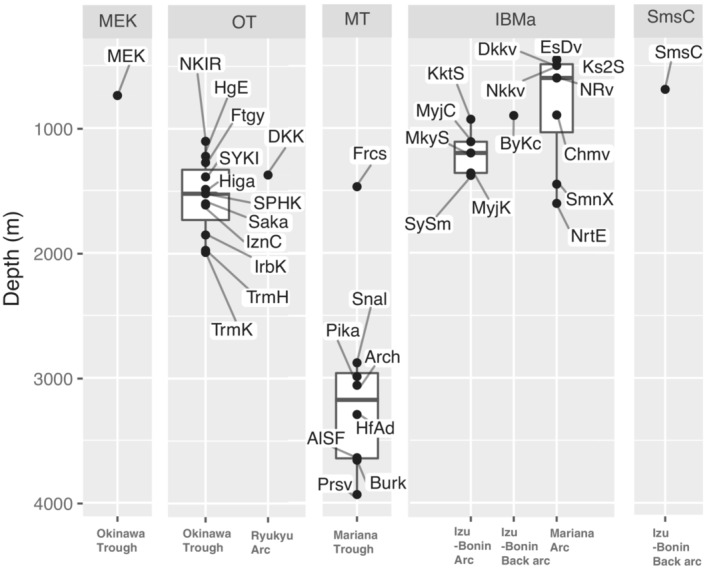
Depth distribution of vent sites separated into modules and subset into their basin.

The presence of Sumisu Caldera as an outlier was detected across all methods. The similarity network disconnected this vent site from the network during the thresholding stage (Figure 2: SmsC) and the SIMPROF results show a divergence of Sumisu Caldera from other vent sites in the Izu‐Bonin‐Mariana before their divergence from the Mariana Trough (Figure 3). Minami‐Ensei Knoll, however, is not considered a clear outlier by the SIMPROF and similarity network methods. Sumisu Caldera and Minami‐Ensei Knoll are the shallowest vent sites of the Izu‐Bonin Arc and Okinawa Trough, respectively, in the present study (Figure 7). The occurrence of Sumisu Caldera as an outlier can be explained, not by its shallow depth, but by its similarity in community composition to cold seeps (Nakajima et al., 2014) likely due to its weak venting activity (Iwabuchi, 1999). The Minami‐Ensei Knoll community, on the other hand, does not resemble a cold seep in its community composition but does have a large proportion of endemic species and a few species uniquely shared with IBMa (Figure 5). Minami‐Ensei Knoll may be distinct within the Okinawa Trough because of its shallow depth (Figure 7) or high methane concentrations relative to the other vent sites in this area (recorded by Chiba (1993) and compared by Nakajima et al. (2014)).

There are minor discrepancies in group membership between the different methods. We prefer the results from the bipartite modularity analysis due to its objectivity compared with similarity network clusters and the additional insights it provides compared with the SIMPROF clusters (Bloomfield et al., 2018). Furthermore, the significant difference of depth, tectonic setting, and broad‐scale dbMEM among the five modules from the MANOVA analysis validates the groups detected by the bipartite modularity analysis, which was not given information on these parameters a priori.

### 4.2 Inter‐module diversity structure

The distinct sub‐regions (excluding Sumisu Caldera and Minami‐Ensei Knoll) detected by the modularity analysis suggest the presence of connectivity barriers within the Northwest Pacific, an area previously classified as a single biogeographic region Bachraty et al., 2009. Studies using this method on the analogous terrestrial ecosystem of island archipelagos (Mullineaux et al., 2018) have also inferred the presence of biogeographic barriers (Carstensen et al., 2012; Kougioumoutzis et al., 2014, 2017). Although we can assume that biogeographic barriers separate the three vent sub‐regions we have detected, the proportion of shared species among them (in the case of OT and IBMa (Figure 5)) and their geographical overlap (in the case of IBMa and MT (Figure 1)) do not support further biogeographic subdivision in the Northwest Pacific region.

By contracting vent site nodes of each module into a single node (Figure 5), it is clear to see which, and how many, species are shared among the distinct sub‐regions. OT shares 12 species with IBMa and none with MT despite both MT and OT being predominantly composed of back‐arc vent sites. The apparent role of IBMa as an intermediary of shared species among sub‐regions (>50% of species is shared with one of the other two sub‐regions) indicates the importance of the spatial arrangement of these modules as IBMa vent sites are mostly found between those of the OT and MT in terms of oceanographic dispersal pathways (Mitarai et al., 2016). The role of IBMa as a regional intermediary between OT and MT is also supported by the relatively high participation coefficient of many of their vent sites (Figure 6). Of the 10 vent sites with the highest participation coefficient, four of them are in the Central/Northern Okinawa Trough and three (Bayonnaise Knoll caldera (ByKc), Myojin Knoll (MyjK), and Myojinsho Caldera (MyjC)) are in the northern Izu‐Bonin Arc. The pathway of the Kuroshio current may act as a bridge for dispersal between the northern Okinawa Trough and northern Izu‐Bonin Arc (Mitarai et al., 2016). Additionally, MyjK and MyjC have particularly high centrality scores due to their position along key pathways connecting the Okinawa Trough and the rest of the similarity network via Fuagoyama (Ftgy) and Daisan‐Kume Knoll (DKK) (Figure 2). Daisan‐Kume Knoll is the only Arc vent site within the Okinawa Trough (Ryukyu Arc) in this study, so it shares both the tectonic setting and a similar depth with Myojin Knoll (Figure 7). However, the occurrence data for Daisan‐Kume Knoll all come from the Gondou field on the western flank of the knoll, which is influenced by both arc and back‐arc tectonic activity Minami & Ohara, 2017. The key pathway connecting the Izu‐Bonin Arc to the Mariana Trough is via a Mariana Arc vent, Northwest Eifuku (NrtE), due to its connection to Forecast (Frcs) (Figure 2). The participation coefficient suggests that Northwest Rota‐1 volcano (NRv) is a more important connection between sub‐regions (Figure 6). This discrepancy is likely due to the centrality measure ignoring “weak” connections removed by the thresholding step. The “strong” connection between NrtE and Frcs may be due to their depth proximity (Figure 7), while the weak connections of NRv and vent sites in the Mariana Trough may be driven by their geographic proximity (Figure 1). Previous studies have suggested that nodes with high centrality in vent similarity networks represent historical stepping‐stones between biogeographic provinces (Kiel, 2016; Moalic et al., 2012). Such historical factors can have a strong role in present‐day biogeography at vents (Kiel, 2017).

Although we were able to detect vent sites that act as important intermediaries among sub‐regions using the similarity network (Figure 2), the bipartite network was not able to detect equivalent “connector nodes” based on Guimerà and Nunes Amaral's (2005a) universal cartographic roles (Figure 6). The lack of vent site connector nodes and the prevalence of periphery nodes in the regional bipartite network is unsurprising considering the large number of module endemics (Figure 5). No species are present in all five modules or even all three sub‐regions. Several species are present in three modules (two sub‐regions and an outlier site). Of particular note is the species *Gandalfus yunohana* (Takeda et al., 2000) (node 28, Figure 5), which is classified as a Network Hub due to its presence in multiple vent sites of OT and IBMa as well as Sumisu Caldera. Additionally, *Enigmaticolus nipponensis* (Okutani & Fujikura, 2000) and *Alvinocaris brevitelsonis* (Kikuchi & Hashimoto, 2000) (nodes 38 and 76, Figure 5) are classified as connector nodes because of their presence in multiple vent sites of OT and IBMa as well as Minami‐Ensei Knoll. These species may have functional traits that allow them to cross these biogeographic barriers through strong dispersal or colonization ability (Economo et al., 2015).

### 4.3 Intra‐module diversity structure

Only the bipartite modularity methods were able to detect the relative importance of vent sites in driving within‐group diversity structure (Figure 6). Based on the shared species as well as geographic and environmental proximity, the sub‐regions may represent three distinct metacommunities of hydrothermal vent species in the region. Regardless of whether they are distinct metacommunities, biogeographic sub‐regions, or even regions, each identified module should be treated as an independent biodiversity management unit (Borthagaray et al., 2018). Thus, the vent sites most important for maintaining network connectivity within their respective modules (highest z_i_ score) should be protected to preserve regional diversity because they may also be key to maintaining gene flow within a metacommunity context. In this context, Sakai (Saka), Alice Springs Field (AlSF), and Nikko volcano (Nkkv) should be prioritized for protection in their respective sub‐regions. Special consideration for conservation should be given to the outlier vent sites of Sumisu Caldera and Minami‐Ensei Knoll due to their high proportion of endemic species (Figure 5). In their study of bird communities on island archipelagos, Carstensen et al. (2012) used the same bipartite network methods to conclude that module hub islands are sources of species for periphery node islands. This conclusion was partly based on the module hub islands being consistently larger in terms of physical area than periphery node islands, a feature we are unable to confirm for hydrothermal vent sites in our study.

There is no evidence for immediate anthropogenic risks, in the form of deep‐sea mining, to the vent sites of the Izu‐Bonin‐Mariana Arc and Mariana Trough, some of which fall within the US Marine National Monument of the Marianas (Menini & Van Dover, 2019). However, the central Okinawa Trough is confirmed as an area of interest for planned hydrothermal vent mining (Okamoto et al., 2019), although specific targets for mining remain publicly unconfirmed. However, the massive sulfide deposits in the Okinawa Trough at Sakai, Izena Cauldron, and Daisan‐Kume Knoll are of particular interest (Ishibashi et al., 2015; Minami & Ohara, 2017). As a “module hub” for the OT sub‐region with the highest within‐module degree, any disturbance to Sakai from mining activity will have a disproportionately strong impact on the linkages among vent sites within the Okinawa Trough metacommunity. The high betweenness centrality of Daisan‐Kume Knoll suggests it plays an important role in sharing species between the Okinawa Trough and Izu‐Bonin Arc; this may be due to its tectonic features that make it an intermediary in terms of local environmental conditions.

### 4.4 Drivers of the diversity structures

The multivariate statistics applied to investigate the drivers of diversity in this study found that the explanatory variables used (depth, tectonic setting, and geodesic distance) explained much of the variation in species distribution data among vent sites (Figure 4) and varied significantly among modules. However, there are likely several other abiotic, biotic and historical covariates that drive regional diversity at the biogeographic and metacommunity scale. The low between‐site variation explained by the environment (depth and tectonic setting) alone is likely due to unknown covariates. Tectonic setting, for example, can influence various parameters such as venting fluid composition, intensity, and stability (Gamo et al., 2013; Mullineaux et al., 2018), which can be drivers of community composition at vents (Juniper & Tunnicliffe, 1997; Kojima & Watanabe, 2015). Furthermore, dissimilarities in venting fluid composition in similar tectonic settings of distinct hydrothermal regions occur because of differences in the local sediment layers, material supply from the subducting slab, and boiling points (Gamo et al., 2013). Although the vent‐obligate species of this study are mostly independent of surface‐derived food, depth is associated with vent community structuring in both this study and previous studies in the Northwest Pacific (Kojima & Watanabe, 2015; Nakajima et al., 2014; Watanabe et al., 2019; Watanabe & Kojima, 2015). Important local controls on community composition can co‐vary with depth, for example, the compositions and concentrations of bio‐relevant chemicals in the vent fluid (Desbruyères et al., 2001). Depth often also co‐varies with water‐mass structure, which is a known biogeographic barrier within the Northwest Pacific Brisbin et al., 2019. Depth can also influence local vent fluid chemistry, such as the boiling point of seawater and the boiling point of venting fluid, and the ensuing phase separation that affects the local chemical composition of such fluids (Gamo et al., 2013). It is difficult to separate the influence of tectonic setting and depth in this region. Although arc and back‐arc vent sites do overlap in terms of depth, particularly between Okinawa Trough and Izu‐Bonin Arc, vent sites that occur on a volcanic arc are characteristically shallower than those that occur on the corresponding back‐arc spreading centre (Figure 7). In general, emissions from vents on volcanic arcs are more acidic and regionally variable than those from back‐arc settings (Resing et al., 2009).

The two dbMEMs explain most of the species assemblage variation and represent their relative geographical position. The calculation of the fine‐scale dbMEM assumes that vent sites in the Okinawa Trough are disconnected from the rest of the regional network while the broad‐scale dbMEM assumes at least indirect connectivity among all vent sites in the Northwest Pacific. It is therefore unsurprising that the broad‐scale dbMEM explains more variation among sites (22% compared with 7%) as it takes into account the species turnover among vent sites of the entire region. Connectivity among vent sites in the Northwest Pacific via larval dispersal is theoretically possible under certain assumptions of larval dispersal behavior (Breusing et al., 2021; Mitarai et al., 2016). This study also demonstrated that dispersal probability was variable among vent sites of the same sub‐regional modules. It is possible that variable dispersal probabilities among vent sites in the Northwest Pacific are responsible for the variation explained by the fine‐ and broad‐scale dbMEM variables. However, both dbMEMs also encompass all spatially autocorrelated variables that we were not able to account for in our analyses (Peres‐Neto & Legendre, 2010). It is therefore likely that the variation explained by these spatial variables represents a dispersal limitation as well as spatially autocorrelated abiotic responses and biotic interactions among species (Thompson et al., 2020). Furthermore, the nature of the dbMEMs assumes that vent sites of a certain geodesic distance from one another can share species and does not take into account the isolation that could be caused by oceanographic or topographic features in the region (Mitarai et al., 2016; Watanabe et al., 2019).

With additional data on relative abundances and/or time series, it would be possible to distinguish the relative roles that biotic, abiotic, and dispersal filters play in structuring the metacommunity (Guzman et al., 2022; Thompson et al., 2020). The uncertainty originating from the multivariate analyses and the lack of available local biotic and abiotic parameters—a common limitation for such remote ecosystems—precludes us from pinpointing the drivers of regional diversity and we simply assume that these critical metacommunity processes are driving the distribution of species. However, the significance of the parameters tested in combination with the structural aspects of the networks allowed us to explore hypotheses of diversity drivers at hydrothermal vents, which could be tested in the future via targeted collections of additional data.

## 5 CONCLUSION

Based on our analyses of species distribution data, we suggest that the Northwest Pacific hydrothermal vent bioregion is divided into three distinct sub‐regions—Okinawa Trough, Izu‐Bonin‐Mariana Arc, and Mariana Trough—that do share some species but have distinct compositions separated by biogeographic barriers. The Minami‐Ensei Knoll and Sumisu Caldera vent sites contain species assemblages dissimilar from all three of the sub‐regions and should be considered outliers that reveal the importance of local environmental factors in distinguishing vent site assemblage structure. Most of the variation in species composition among individual vent sites and the sub‐regions can be explained by local environmental filters associated with tectonic setting and depth, as well as the geodesic distance among vent sites. Future investigations into local environmental filters for hydrothermal vent communities are necessary to evaluate their role in structuring the metacommunity.

The shared species and linkages across the distinct sub‐regions of the Northwest Pacific can be disproportionately attributed to a small number of vent sites, namely Daisan‐Kume Knoll, Myojin Knoll, Northwest Eifuku, and Forecast. Disturbances to these nodes with high centrality would have relatively strong impacts on maintaining connectivity among the three sub‐regions, as they are key nodes in preventing the collapse of the regional network (Thompson et al., 2015). The vent sites most important for linking within the sub‐regions of the Okinawa Trough, Izu‐Bonin‐Mariana Arc, and Mariana Back‐arc are Sakai, Nikko Volcano, and Alice Springs Field, respectively. These three vent sites play the most important role in maintaining biodiversity in the Northwest Pacific, on time scales pertinent to conservation, by connecting the sub‐regional metacommunities through shared species. Additional biological data are required to disentangle the relative roles of local environmental filtering, biotic interactions, and dispersal in structuring the metacommunity at the regional scale. However, the network approach of this study enables us to indicate key vent sites for maintaining regional diversity based on species distribution data; this is a powerful tool for informing the conservation of this remote and vulnerable ecosystem through spatial management strategies in the age of deep‐sea mining.

## AUTHOR CONTRIBUTIONS

**Otis Davey Brunner:** Conceptualization (equal); data curation (supporting); formal analysis (lead); methodology (lead); writing – original draft (lead); writing – review and editing (equal). **Chong Chen:** Conceptualization (supporting); data curation (equal); writing – review and editing (equal). **Thomas Giguère:** Data curation (equal); writing – review and editing (equal). **Shinsuke Kawagucci:** Data curation (equal); writing – review and editing (equal). **Verena Tunnicliffe:** Conceptualization (supporting); data curation (equal); supervision (supporting); writing – review and editing (equal). **Hiromi Kayama Watanabe:** Conceptualization (supporting); data curation (equal); writing – review and editing (equal). **Satoshi Mitarai:** Conceptualization (equal); supervision (lead); writing – review and editing (equal).

### OPEN RESEARCH BADGES

This article has earned Open Data, Open Materials and Preregistered Research Design badges. Data, materials and the preregistered design and analysis plan are available at [[insert provided URL(s) on the Open Research Disclosure Form]].

## Supporting information


Appendix S1

## Data Availability

All data used in this study are available within the supplementary tables in the form of excel spreadsheets.
